# Breastfeeding and childhood cancer risks: OSCC data

**DOI:** 10.1038/sj.bjc.6600881

**Published:** 2003-04-01

**Authors:** R J Lancashire, T Sorahan

**Affiliations:** 1 Department of Public Health and Epidemiology, University of Birmingham, Edgbaston, Birmingham B15 2TT, UK; 2 Institute of Occupational Health, University of Birmingham, Edgbaston, Birmingham B15 2TT, UK

**Keywords:** breastfeeding, childhood cancer

## Abstract

Matched pair analysis relating to 3376 mothers of children who died of cancer 1972–1981 and of healthy control children from the Oxford Survey of Childhood Cancers showed no evidence of protection from breastfeeding for acute lymphocytic leukaemia (OR 1.04, 95% CI 0.86–1.26), for all cancers combined (OR 1.04, 95% CI 0.93–1.15) or for other groupings. Analyses by duration of breastfeeding also failed to support the protective hypothesis.

The UK Childhood Cancer Study (UKCCS) has reported weak evidence of a protective effect of breastfeeding for childhood cancers overall (Beral et al, 2001). It was concluded that breastfeeding may well offer a modest protective effect on childhood cancers overall or that some systematic bias was operating in the majority of the published studies. No findings were available from the Oxford Survey of Childhood Cancers (OSCC) on breastfeeding and childhood cancer risks; these are now provided.

## MATERIALS AND METHODS

The OSCC, a national case–control study into the aetiology of childhood cancer, began in Oxford in 1955, but has been located at the University of Birmingham since 1975 (Stewart et al, 1958; Gilman et al, 1988). The survey sought to interview the parents (usually the mother) of all children dying of cancer before their 16th birthday in England, Wales and Scotland for the period 1953–1984. A number of standard questionnaires, covering a wide range of social and medical topics, were used during the course of this prolonged study. Data on breastfeeding in infancy were not collected for all years of the study, but had been sought for 1972–1981 deaths and matching controls.

There were 7590 childhood cancer deaths in England, Wales and Scotland for the period 1972–1981. Interview data had been obtained from the parents of 4288 (56%) of these children. Parents of 1261 case children had refused to participate with the survey, a further group of 771 case parents had moved abroad or to an unknown address, and the remaining 1270 case parents had not replied to survey requests (the large majority), their general practitioner had advised the survey not to approach them or arrangements for interviews had fallen through. The response rate from case parents approached was thus at least 63% (4288/(7590−771)).

For each case child with interview data, a ‘control list’ of six children, matched for sex and date of birth, was selected from the birth register of the local authority area in which the case child died. Control parents were contacted in turn until one control family agreed to be interviewed. Interview data were obtained for 3827 control children (2078 first choices, 690 second choices and 1059 later choices). (Control interviews were not obtained for 461 case children with interview data; these cases do not feature in these analyses.) Only 54% of first choices may seem a low percentage, but the birth registers from which the controls were selected had been compiled, on average, some 8 or 9 years before the interviews were arranged. The case and control parents within each pair were interviewed by the same person, usually a physician or nurse from the local health authority.

For the purpose of this report, the interview folders of all matched pairs were reviewed and information on breastfeeding in infancy was reabstracted and amalgamated with existing study computer files. The main interview questionnaire included the following questions: ‘Did you breastfeed’ and ‘For how long’. When the duration of breastfeeding was reported with upper and lower values, both values were computerised, but the upper value was selected for the purpose of this analysis.

Matched pairs in which the case child was adopted (*n*=40) were excluded, as were 13 cases for which hospital records indicated that the death certificate diagnosis of cancer was incorrect. It was not possible to exclude adopted controls. Matched pairs in which the case child died in the first year of life (*n*=242) or in which the first symptoms of a later fatal disease occurred in the first nine months of life (*n*=156) were excluded as these matched pairs might only show the influence of cancer on breastfeeding rather than the converse. Case and control data relating to infancy breastfeeding (3376 matched pairs) were then compared by means of conditional logistic regression using the EGRET program. Breastfeeding history was also analysed in conjunction with maternal age at the birth of the child, social class based on paternal occupation and sibship position of the child. A standard classification (Office of Population Censuses and Surveys, 1970) had been used to code social class, except that members of the Armed Forces were classified as social class III and a maternal occupation was used when a paternal occupation was unavailable. The purpose of these last analyses was to allow for the effects of other variables, so that the independent effects of breastfeeding history could be examined. The odds ratio was used to obtain estimates of relative risk. Risks are shown relative to a baseline risk of unity for children who were never breastfed.

Analyses were also carried out using unconditional logistic regression so that comparisons with each of the series of cancer subtypes (leukaemia, *n*=1342; other reticuloendothelial cancers, *n*=326; all other cancers, *n*=1108) could be made with the entire series of controls (*n*=3376). These analyses adjusted for sex of child, age at death or corresponding age for control child (1–4, 5–9, 10–14 years), social class, sibship position and maternal age at birth of child. Computerised information on region of residence was not available for these latter analyses.

## RESULTS

Number of cases and controls are shown by categories of breastfeeding history (maternal reports) in Table 1

**Table 1:** Childhood cancer risks in relation to breastfeeding in infancy, OSCC data, 1972–1981 deaths

**Breastfeeding history**	**Cases**	**Controls**	**OR**^a^	**(95% CI)**	**O**R^b^	**(95% CI)**	**OR**^c^	**(95% CI)**
*Acute lymphocytic leukaemia (ALL)* (*n*=*948*)								
Never	480	490	1.0				1.0	
Ever breastfed	461	452	1.04	(0.86–1.26)	0.99	(0.82–1.20)	1.05	(0.90–1.21)
Breastfed								
<1 month	151	137	1.15	(0.87–1.51)	1.10	(0.83–1.46)	1.16	(0.93–1.43)
1–6 months	241	246	1.01	(0.81–1.26)	0.96	(0.77–1.20)	0.96	(0.80–1.15)
⩾7 months	62	66	0.97	(0.66–1.43)	0.90	(0.60–1.34)	1.09	(0.80–1.48)
Unknown duration	7	3	—					
Not known	7	6	—					
*P*-value for trend^d^			*P*=1.00	*P*=0.55	*P*=0.99			
								
*Other leukaemia* (*n*=394)								
Never	205	208	1.0				1.0	
Ever breastfed	187	181	1.06	(0.78–1.45)	1.03	(0.74–1.43)	0.99	(0.80–1.23)
Breastfed								
<1 month	51	40	1.29	(0.82–2.04)	1.24	(0.77–1.98)	0.95	(0.68–1.32)
1–6 months	105	114	0.94	(0.66–1.35)	0.90	(0.62–1.31)	0.97	(0.76–1.25)
⩾7 months	30	26	1.16	(0.65–2.07)	1.16	(0.64–2.11)	1.17	(0.77–1.77)
Unknown duration	1	1	—					
Not known	2	5	—					
*P*-value for trend^d^			*P*=0.81	*P*=0.92	*P*=0.71			
								
*Leukaemia* (*n*=1342)								
Never	685	698	1.0				1.0	
Ever breastfed	648	633	1.05	(0.89–1.23)	1.00	(0.85–1.18)	1.03	(0.91–1.18)
Breastfed								
<1 month	202	177	1.18	(0.93–1.50)	1.14	(0.89–1.45)	1.10	(0.91–1.33)
1–6 months	346	360	0.99	(0.82–1.19)	0.95	(0.79–1.15)	0.97	(0.83–1.13)
⩾7 months	92	92	1.03	(0.75–1.43)	0.98	(0.71–1.37)	1.11	(0.85–1.44)
Unknown duration	8	4	—					
Not known	9	11	—					
*P*-value for trend^d^			*P*=0.90	*P*=0.70	*P*=0.84			
								
*Other reticuloendothelial cancers* (*n*=326)^e^								
Never	160	182	1.0				1.0	
Ever breastfed	163	142	1.34	(0.97–1.87)	1.32	(0.93–1.89)	1.10	(0.87–1.39)
Breastfed								
<1 month	56	49	1.35	(0.85–2.13)	1.38	(0.86–2.25)	1.28	(0.92–1.77)
1–6 months	84	74	1.37	(0.92–2.06)	1.33	(0.86–2.06)	0.99	(0.75–1.31)
⩾7 months	23	18	1.53	(0.78–3.00)	1.56	(0.75–3.21)	1.23	(0.77–1.97)
Unknown duration	0	1	—					
Not known	3	2	—					
*P*-value for trend^d^			*P*=0.07	*P*=0.11	*P*=0.62			
								
*All other cancers* (*n*=*1708*)								
Never	901	901	1.0		1.0		1.0	
Ever breastfed	777	797	0.98	(0.84–1.13)	0.95	(0.82–1.11)	0.97	(0.86–1.10)
Breastfed								
<1 month	228	236	0.97	(0.79–1.20)	0.94	(0.76–1.17)	0.98	(0.82–1.17)
1–6 months	441	457	0.97	(0.81–1.15)	0.94	(0.79–1.12)	0.97	(0.84–1.12)
⩾7 months	105	101	1.04	(0.77–1.41)	1.06	(0.78–1.45)	0.96	(0.74–1.23)
Unknown duration	3	3	—					
Not known	30	10	—					
*P*-value for trend^d^			*P*=0.96	*P*=0.85	*P*=0.57			
								
*All childhood cancers* (*n*=3376)								
Never	1746	1781	1.0		1.0		1.0	
Ever breastfed	1588	1572	1.04	(0.93–1.15)	1.01	(0.91–1.12)	1.01	(0.91–1.11)
Breastfed								
<1 month	486	462	1.08	(0.93–1.25)	1.05	(0.90–1.22)	1.05	(0.91–1.22)
1–6 months	871	891	1.00	(0.89–1.13)	0.98	(0.87–1.11)	0.97	(0.87–1.09)
⩾7 months	220	211	1.07	(0.87–1.32)	1.06	(0.85–1.31)	1.04	(0.85–1.27)
Unknown duration	11	8	—					
Not known	42	23	—					
*P*-value for trend^d^			*P*=0.55	*P*=0.77	*P*=0.94			

a Obtained from conditional logistic regression applied to matched pairs.

b Obtained from conditional logistic regression applied to matched pairs with additional adjustment for social class based on paternal occupation (five levels: I, II, III, IV, V), age of mother at birth of survey child (six levels: <20, 20–24, 25–29, 30–34, 35–39, ⩾40 years), and sibship position (five levels: 1, 2, 3, 4, ⩾5).

c Obtained from unconditional logistic regression using all controls for comparison group, adjusted for sex of child, age at death (or corresponding age for controls) (1–4, 5–9, 10–14 years), social class, maternal age at birth of child and sibship position.

d *P*-value for trend across four levels of breastfeeding history variable (never, <1, 1–6, ⩾7 months) levels scored 1–4.

e Comprising lymphosarcoma, Hodgkin's disease, reticulosarcoma, giant follicular lymphoma, storage reticulosis, histiocytic reticulosis, chloroma and Burkitt's lymphoma.

, together with corresponding odds ratios. These odds ratios are shown for matched pair analyses first unadjusted for other variables (left-hand column) and then adjusted for the variables described previously (middle column). Odds ratios obtained for unconditional logistic regression are shown in the right-hand column. There was no suggestion of a protective effect from infancy breastfeeding either for acute lymphatic leukaemia, other leukaemia, other reticuloendothelial cancers, all other cancers combined, or for all cancers combined. For all categories of childhood cancer shown in the table, there were no significant positive or negative trends with duration of breastfeeding.

## DISCUSSION

The study was relatively large but was dependent on self-reported histories. It is also possible that the deaths of the case children influenced the information supplied by case mothers. In addition, participation rates in the later phases of the OSCC were modest. There is thus scope for biased comparisons of cases and controls to have been made and for such bias to have been introduced in a number of different ways. Nevertheless, this study has failed to support the hypothesis that breastfeeding protects against childhood leukaemia or other childhood cancers
